# Plant Biotechnology—An Indispensable Tool for Crop Improvement

**DOI:** 10.3390/plants13081133

**Published:** 2024-04-18

**Authors:** Ranjith Pathirana, Francesco Carimi

**Affiliations:** 1 School of Agriculture, Food and Wine, Waite Campus, University of Adelaide, Urrbra, SA 5064, Australia; 2 Istituto di Bioscienze e BioRisorse (IBBR), Consiglio Nazionale delle Ricerche, Via Ugo la Malfa, 153, 90146 Palermo, Italy; francesco.carimi@ibbr.cnr.it

## 1. Introduction

**Figure 1:**
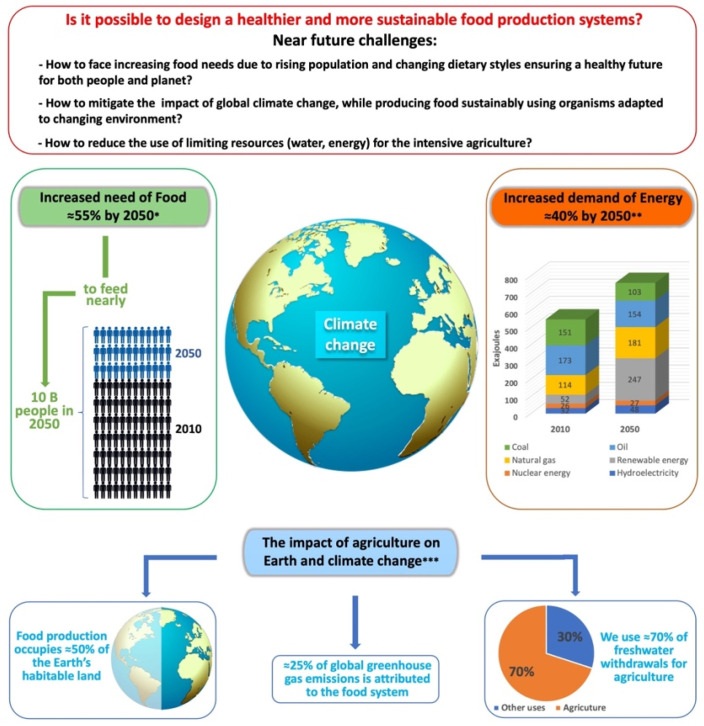
Food systems are increasingly vulnerable due to human pressures on natural ecosystems and the climate: The challenge ahead. * World Resource Institute (WRI)’s 2023 report. Available online: https://research.wri.org/wrr-food (accessed on 22 January 2024); ** Statista Energy Consumption Worldwide from 2000 to 2019, with a forecast until 2050, by Energy Source. Available online: https://www.statista.com/statistics/222066/projected-global-energy-consumption-by-source/ (accessed on 22 January 2024); *** Mahpul IN, Mohamad AH, Mazalan MF, Razak A, Rasyidee A (2021) Population, food security, nutrition and sustainable development. Available online: https://www.un.org/development/desa/dpad/publication/un-desa-policy-brief-102-population-food-security-nutrition-and-sustainable-development/ (accessed on 22 January 2024) [4].

Traditional plant breeding has helped to increase food production dramatically over the past five decades, and many countries have managed to produce enough food for the growing population, particularly in the developing world. Sustaining these gains in crop productivity and adapting to climate change are becoming urgent concerns in modern times. In fact, yield increases in our major cereals have slowed down in the past 20 years. Global hunger is still above pre-pandemic levels, with around 690–783 million people faced with hunger in 2022, and meeting Sustainable Development Goal 2 of ending hunger by 2030 has become a daunting task [1]. Although increased yields through the Green Revolution helped to cultivate an additional 18–27 million hectares, this increased food production was accompanied by environmental degradation and micronutrient deficiencies across populations [2,3]. Developing crop cultivars that meet the present-day requirements of agriculture and horticulture is challenging, as they need to provide sustainable food and healthful nutrition for populations, and, at the same time, must be environmentally friendly and resilient to climate change. The global community is projected to face increasing food crises due to changing dietary styles and the rising population, which is set to reach almost 10 billion people by 2050 [4]). The challenge ahead is determining how to reduce the use of limiting resources (water, energy, and agricultural land) for intensive agriculture, ensuring sufficient production of food (Figure 1). Taking even the most conservative estimates, food production needs to double in the coming 30 years to meet the basic demands of the growing population [5]. Despite these challenges, there is growing evidence that food security and adequate nutrition for the global population can be achieved using climate-smart, sustainable agricultural practices, while reducing the negative impacts of agriculture on the environment, particularly greenhouse gas emissions [6].

Plant biotechnology is seen as the breakthrough technology that can help to meet this challenge in this next phase of plant breeding. Plant biotechnologies that aid in developing new varieties and individual traits within existing plant varieties include cell and tissue manipulation, marker-assisted selection, transgenic technologies, genomics, and molecular breeding. Cell and tissue culture technologies provide a range of applications in the creation, conservation, and utilization of the genetic variability in crops, such as in vitro pollination and embryo rescue for distant hybridization, the production of haploids and doubled haploids, polyploid breeding, in vitro mutagenesis, somaclonal variation, in vitro selection, germplasm preservation (in vitro for medium-term and cryopreservation for long-term), protoplast fusion for producing somatic hybrids, and gene manipulation for producing transgenic crops or the newly emerging techniques that allow for the generation of gene-edited plants.

**Figure 2:**
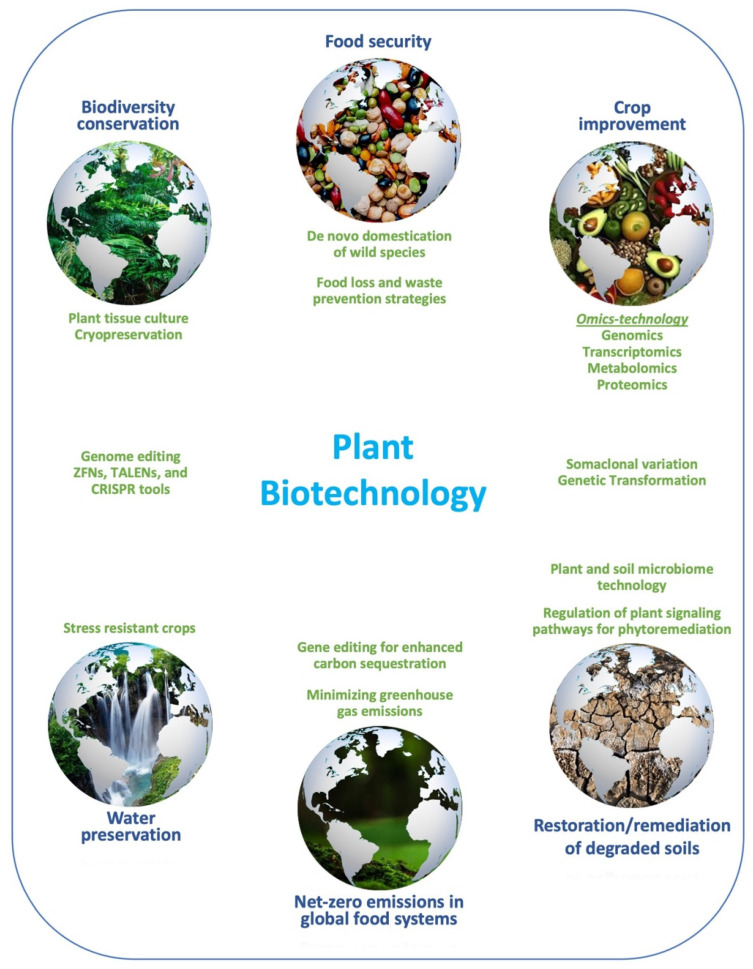
The many aspects of the contribution of biotechnology towards crop improvement for resilient food systems while also contributing to environmental protection.

High-resolution genetic analysis has allowed physical mapping and positional gene cloning for traits of interest, while molecular markers allow for the characterization of germplasm and finding duplicates and gaps in collections [2]. They are becoming indispensable in some breeding programs when used for the early culling of unwanted material in perennial crops such as in the case of culling male vines early in hybrid populations, screening in kiwifruit for marker-assisted selection [7,8], the development of saturated linkage maps, and pyramiding genes in introgressive breeding [9,10]. Despite the strict laws governing genetically modified crops, transgenic varieties of maize, soybean, rapeseed, cotton, tomato, potato, papaya, etc., occupy over 190 million hectares across 26 countries, grown by 17 million farmers, bringing in both economic and environmental benefits and, at the same time, some social controversy [6]. The many tools that plant biotechnology provides for crop improvement for developing resilient food systems while conserving the environment are shown in Figure 2. These aspects have been addressed in the 17 papers published in this Special Issue titled ‘Plant Biotechnology and Crop Improvement’. There have been four general review papers covering different biotechnologies and thirteen original research contributions focusing on different crop groups, including tropical and temperate cereal, legume, root and tuber, fruit, ornamental, and industrial crops. With 44,000 views and 86 citations at the time of writing, this Special Issue has attracted much attention across the scientific community as expected, considering the relevance of the topic to the current challenges in global food production.

This Special Issue also contains methodology development for plant genetic transformation through the use of an easy selection marker for discriminating transformed plants from escapes. The reviews in this Special Issue look at specific trait improvements such as stress resistance using gene-editing technologies and manipulation of phytohormone metabolism, as well as exploring if a second “quantum-leap” in food production is possible using these technologies. Another review describes the contribution of genomics to our understanding of crop evolution. With a wide array of applications of plant biotechnology to crop improvement available, the research papers addressed several of these technologies in extensively cultivated crops such as wheat, barley, bean, and potato, as well as in underutilized crops with high potential such as tef and prickly pear. The technologies applied to improve crops in these papers include somaclonal variation, induced mutagenesis, in vitro polyploidization, embryo rescue, and gene editing. Several papers describe the use of genomics, transcriptome analysis, molecular markers, and metabolic profiling to assist in selection in breeding programs and monitoring of transgenic plants.

The possibility of modifying phytohormone metabolism and signaling is a promising direction of research aimed at the improvement of crop productivity and stress tolerance. In her review, Nowicka (contribution 1) summarizes the state-of-the-art research concerning the modulation of phytohormone content aimed at the stimulation of plant growth and the improvement of stress tolerance. In particular, the roles of auxins, cytokinins, gibberellins, brassinosteroids, abscisic acid, ethylene, jasmonic acid, and their derivatives are analyzed. The author hypothesizes that modification of this signaling at various levels, from elements of signaling cascades, through transcription factors to miRNAs, is a very promising direction of genetic engineering of crop plants aimed at improving the resilience of plants.

Remarkable progress in genome-editing technologies has been achieved over the past 10 years and have begun to show extraordinary utility to develop crop varieties with superior qualities, or those that can tolerate adverse environmental conditions. In their review, Hamdan et al. (contribution 2) provide a detailed analysis of the genome-editing technologies that have been expertly applied to improve important agronomic traits, especially yield, quality, and stress resistance of the most important crops. In particular, the review focuses on the Clustered Regularly Interspaced Palindromic Repeats (CRISPR/Cas) system, which has been the focus in recent years as a revolutionary genome-editing tool used for various crops. The authors discuss the current developments and future applications of genome-editing technologies for developing crops that can help in mitigating the impacts of climate change on agriculture with notes on future perspectives. A bibliographic analysis is also presented covering CRISPR-related papers published from 2012 to 2021 (10 years) to identify trends and potential in CRISPR/Cas-related plant research. The authors conclude that combining conventional and more innovative technologies in agriculture would be the key to optimizing crop improvement beyond the limitations of traditional agricultural practices. A more pessimistic view is provided in a review carried out by Buzdin et al. (contribution 3), reporting that, according to estimates, global crop yields must double by 2050 to adequately feed an increasing global population without a large expansion of crop area. To achieve this “quantum-leap” in improvements in crop yield, we must respect environmental constraints and, at the same time, reduce the impact of agriculture on the environment. The authors support the long-debated idea that new technologies are unlikely to provide a rapidly growing population with significantly increased crop yield. Finally, in their review, Zhao et al. (contribution 4) analyze how recent advances in genomics have revolutionized our understanding of crop domestication. The authors summarize cutting-edge research on crop domestication by presenting the main methodologies and analyze the prospects for both targeted re-domestication and de novo domestication of wild species.

## 2. Cereal Crops

Dramatic increases in rice and wheat yields were achieved during the ‘Green Revolution’, where dwarfing genes were transferred to adapted cultivars through crossbreeding. The ‘Green Revolution’, with its high-input and technology-dependent approach, has been able to feed the growing world population in recent decades. It ensured food security, particularly in developing nations. However, long-term impacts are now evident: degraded soils, reduced groundwater levels, contaminated and salinized water bodies, and reduced biodiversity. Furthermore, high crop yields cannot be sustained without increased fertilizer use [6,11,12,13]. Traditional crossbreeding is straightforward when selecting for morphological traits that are easy to observe in field, such as height, grain size, color, and leaf shape, etc. The main change in rice and wheat achieved during the “Green Revolution” is dwarfing, resulting in greater partitioning of photosynthates in grains and better fertilizer response, without lodging. Hence, it was not difficult to identify dwarf plants in the segregating populations. However, traits such as nutritional quality, disease, and abiotic stress resistance are not easy to select visually in segregating populations under field conditions where breeders encounter many variables. Lab-based approaches to increase genetic variability or to genetically modify and select desirable genotypes are therefore required.

Over the past few decades, biotechnology has made significant contributions to cereal crop improvement by enhancing yield, nutritional content, biotic and abiotic stress tolerance, herbicide tolerance, and many other valuable traits. It has also played a crucial role in promoting environmental sustainability and has had positive economic impacts on agriculture. For example, the introduction of perennial cereals can alleviate many problems of annual monocultures [6,11,12,13]. *Thinopyrum* spp. is the most sought-after perennial grain in hybridization programs with wheat as it hybridizes freely with *Triticum*, producing fertile progeny [13], and perennial selections have outperformed the standard wheat cultivars in grain protein and mineral nutrient contents [14]. Yet, with genomic tools, selection for perennial growth and other quality traits would be easier and faster [13]. Thus, intermediate wheatgrass has been used in sequencing and marker-assisted recurrent selection [15], and a high-quality genetic map is now available online [16]. With these developments, breeding perennial wheat for large-scale cultivation will be possible.

Similarly, perennial rice (PR) will be the start of a second ‘Green Revolution’ as the data from 15,333 ha of perennial rice grown by 44,752 small holder farmers in southern China demonstrate [17]. The parents for the breeding program to develop PR were ‘RD23’, a cultivar of *Oryza sativa* ssp. *indica*, and a rhizomatous and perennial African species, *O. longistaminata*. Embryo rescue (a tissue-culture-based biotechnological intervention) of F_1_ facilitated overcoming incompatibility and resulted in the foundation material for developing the commercialized PR. PR produced similar yields to annual rice over a period of four years, with eight harvests from a single planting. Farmers prefer PR due to 58.1% labor savings and 49.2% savings on inputs every growth cycle. Higher organic carbon and nitrogen accumulation in soils and improved soil water retention are other advantages [17]. Attempts to develop perennial rye using perennial wild rye *Secale montanum* L. [13] and perennial maize using tetraploid maize (*Zea mays* 2n = 4x = 40), tetraploid *Tripsacum dactyloides* (2n = 4x = 72), and tetraploid *Z. perennis* (2n = 4x = 40) [18] are underway.

Many other biotechnological interventions are possible in the development and selection of climate-resilient cereals. For example, Kruglova and Zinatullina [19] describe many examples of in vitro selection for drought, simulating water deficiency in culture media. They suggest using embryos at a certain developmental stage, when they are autonomous. In vitro selection for iron toxicity [20], aluminum toxicity [21], nickel, and NaCl toxicity tolerance [22] has been demonstrated in cereal crops. In vitro mutation induction and selection have also been demonstrated in many cereals [23]. More targeted mutations can be used in crop improvement thanks to the development of techniques such as Targeting Induced Local Lesions in Genomes (TILLING), as well as the latest gene-editing techniques. For example, Acevedo-Garcia et al. [24] developed bread wheat cultivars resistant to powdery mildew by TILLING. The first genome-editing tools were Zinc Finger Nucleases (ZFN) and Transcription Activator-Like Effector Nucleases (TALENs), but, later, Clustered Regularly Interspaced Short Palindromic Repeats (CRISPR) and Crispr associated protein (Cas) became the most widely used genome-editing tool due to its high editing efficiency, multiplex capability, and ease of use. Gene editing has enabled researchers to increase grain number and size in rice, and grain weight and yield in wheat. Powdery mildew resistance in wheat and resistance to *Xanthomonas* in rice have also been achieved using gene-editing technologies [25].

Wheat, barley, and tef are among the cereal crops covered in this Special Issue. In wheat breeding, crested wheatgrass *Agropyron cristatum* is considered a potential donor of valuable traits for abiotic (cold, drought, and salinity) and biotic (leaf rust, stripe rust, and powdery mildew) resistance. Crested wheatgrass belongs to the tribe Triticeae to which wheat also belongs and similar to *Triticum* has polyploid series with a basic chromosome number x = 7, but with the basic genome *P. Agropyron* is in the tertiary gene pool in the context of wheat breeding, and phylogenetically more distantly related than those in the primary and secondary gene pools of wheat. Fortunately, previous genetic studies have revealed that synteny is conserved between wheat and the P genome. Being a perennial species widely used in temperate regions for grazing beef and dairy cattle, it is also a candidate for transforming wheat into a perennial crop in futuristic sustainable agricultural systems [26]. In hybrids of these two species, chromosome recombination is key to transferring beneficial alleles from crested wheatgrass to wheat. Using in situ hybridization, a technique used to locate specific genomic DNA sequences within chromosomes, Prieto et al. (contribution 5) analyzed chromosome associations during meiosis in *Triticum aestivum* lines carrying chromosome introgressions of breeding interest (5P and 6P) in two sets of progenies: those with and without the *Ph1* locus located in the long arm of chromosome 5B of wheat, known to genetically control chromosome pairing and recombination. The authors did not find homoeologous chromosome pairing either in the presence or absence of the *Ph1* locus, indicating that this locus does not influence chromosome pairing between the two species.

The second cereal featured in this Special Issue is barley. Co-evolution of *Hordeum vulgare* and the fungus *Blumeria graminis* (D.C.) Golovin ex Speer, f. sp. *hordei* Em. Marchal (*Bgh*), causing powdery mildew is well studied and recorded, with more than 70 resistance genes. Most of the cultivated winter barley varieties carry one or more of these genes in different combinations, and this information can be used to authenticate accessions in a collection. Using sets of five single-plant progenies (SPPs) per accession from 172 winter barley accessions belonging to the core collection of Czeck gene bank, Dreiseitl and Nesvadba (contribution 6) tested 53 isolates of the pathogen for virulence/avirulence. While the majority of the accessions showed a single phenotype for resistance in their five SPPs, 78 (45.7%) accessions had more than one phenotype indicating heterogeneity in their seed stocks. With defined powdery mildew resistance genes in the SSPs, these accessions can be used with confidence in barley breeding for powdery mildew resistance. The third cereal featured in this Special Issue is the ancient grain tef (*Eragrostis tef* (Zucc.) Trotter), an underutilized cereal from Ethiopian highlands with outstanding nutritional value and more resilient than traditional cereals under marginal conditions. With no gluten epitopes, it is recommended for people suffering from celiac disease; hence, it is gaining increased attention around the globe. In this Special Issue, Numan et al. (contribution 7) describe the use of in vitro culture and mutagenesis to improve disease and lodging resistance, as well as the use of molecular markers for selection in tef. They conclude by discussing the potential of genome-editing technologies in tef improvement.

## 3. Pulse Crops

Pulses constitute an integral part of cropping systems and provide low-cost proteins in diets as well as essential micronutrients. They are the primary source of proteins in vegetarian and vegan diets as well as in the diets of the majority of the population in many developing countries where protein malnutrition is widespread. They improve soil through biological nitrogen fixation, helping to reduce nitrogen fertilizer requirements of the pulse crop, as well as for the next non-legume crop in cropping systems. The value of pulses was highlighted by declaring 2016 as the year of pulses at the 68th United Nations General Assembly (UNGA) with Food and Agriculture Organization (FAO) facilitating its implementation with the participation of Governments and various other stakeholders [27]. Recognizing the potential of pulses to achieve the 2030 Agenda for Sustainable Development, the UNGA designated 10 February 2023 as World Pulse Day [28]. Additionally, legumes are an important component of animal feed.

Conventional breeding of leguminous crops has been based on the selection for agronomic traits in the vegetative and reproductive phases that have distinct heritability values. One of the main features for mechanized cultivation of legumes is their transformation from an indeterminate growth habit to a determinate growth habit, facilitating synchronous flowering, pod maturation, and resistance to lodging. Soybean yields have increased globally from around 1130 kg ha^−1^ in the early 1960s to the current 2800 kg ha^−1^, with the yields in the three top soybean-producing countries (USA, Brazil, and Argentina) recording 3200–3300 kg ha^−1^ [29]. The breeding of determinate cultivars is a major factor for such yield increases and the expansion of the production area through mechanization. The determinate trait is recessive and monogenically inherited, with the heterozygous individuals showing semi-determinate growth [30,31]. Determinate growth habits have been bred into many other leguminous crops used for seeds, such as pea, chickpea, pigeon pea [31,32], mung bean, black gram [33], grass pea, and cowpea [34]. Many of the first determinate cultivars were bred by mutation induction [35] and not through traditional crossbreeding.

Pulses were regarded as ‘orphan crops’ until recently due to lesser attention given to them compared to cereals. However, many of the pulse crops have now become ‘mainstream crops’, with draft genomes of many of them completed in the past decade [36,37,38,39] improving the efficiency of breeding efforts. Next-generation sequencing technologies have enabled the deployment of modern genomic tools, including a range of molecular markers associated with many agronomic traits, and disease and abiotic stress tolerances [40].

Beans, chickpeas, and peas are the most well-known and widely consumed pulses in the world [28], and two of these are featured in this Special Issue. Common beans (*Phaseolus vulgaris*) were introduced to Ethiopia in the 16th century, and farmers have selected varieties adapted to the local climate and soils over centuries. Their wide genetic diversity, particularly their tolerance to biotic and abiotic stress, has been incorporated in selections developed by the National Common Bean Improvement Program in Ethiopia. Tigist et al. (contribution 8) used 144 genotypes in a multilocation study to understand the variation in 15 agro-morphological traits. Multivariate analysis revealed six principal components. Based on agro-morphological traits, the clustering patterns were according to seed size with considerable genetic variation for the studied characters. The study revealed several accessions with distinct advantages in terms of agro-morphological traits and adaptability suitable for further improvement in the breeding program.

Chickpea (*Cicer arietinum*) is the second most consumed pulse after dry beans, and Australia is a major producer and exporter of this pulse. Among all the continents, Australia is the second driest continent after Antarctica; hence, the drought resistance of crops is a top priority in breeding programs. In both Australia and India (the largest producer of chickpea), chickpea is sown on residual summer moisture and left to grow in progressively depleting soil water, finally maturing under terminal drought. There are many traits associated with drought tolerance, such as root biomass and some leaf anatomical and physiological features. Early maturity is a drought escape strategy in crops such as chickpea sown on residual moisture. There is intensive ongoing work in identifying molecular markers for marker-assisted selection for drought tolerance in chickpea, and a quantitative trait loci (QTL) hotspot region for this trait has been found. The variety ‘Geletu’ with a high yield and drought tolerance was released in 2019 through a backcrossing program to introgress drought tolerance from accession ICC4958 to a high yielding Indian cultivar ‘JG11’, using this hotspot as a selection marker in a backcross breeding program [41]. However, other more innovative methods for screening populations for drought tolerance in the early growth stages would further accelerate breeding. In their paper in this Special Issue, Purdy et al. (contribution 9) went a step further and identified metabolites in young, watered seedlings of chickpea that can be prognostically used to predict seed numbers in mature plants under terminal drought. Among the yield components of annual crops, it is the seed number, not the seed size (weight) that is sacrificed under abiotic stress, drought in particular (contribution 9) [42,43]. Hence, identifying metabolites that can be used as indicators of seed number under terminal drought later in the life cycle would help in selecting drought-tolerant segregants early on in breeding populations. In chickpea, pinitol, sucrose (negative correlation with seed number), and gamma-aminobutyric acid (positive correlation) can be used to predict high or low seed numbers under these conditions (contribution 9). This is the first instance where a predictive marker was identified for screening drought tolerance that could be used by breeders to identify genotypes that perform well under adverse conditions, without having to expose them to drought.

## 4. Root and Tuber Crops

Almost all root and tuber crops are traditionally propagated vegetatively, and most are either sterile or partially sterile (cassava and yam); moreover, flowering is irregular and asynchronous (cassava), or crops do not flower at all (aroids such as *Colocasia*) [44]. Therefore, these crops are ideal candidates for improvement through in vitro-based biotechnological approaches. Potato [45] and sweet potato [46] have been improved through hybridization and selection; therefore, modern genomics tools are invaluable in improving the efficiency of breeding.

As tuber and root crops are an important source of carbohydrates in many impoverished communities around the world, attention has been focused on improving their mineral and vitamin contents because hidden hunger resulting from their deficiencies is prevalent in these communities, with an estimated two billion people affected [47]. About 800 million people use cassava (*Manihot esculenta* Crantz—Euphorbiaceae) as their staple food, and one third of the sub-Saharan population depends on cassava for over 50% of their caloric intake [48]. Breeding for increased mineral nutrition in cassava is hampered by the lack of genetic variation for these traits [49]; hence, transgenic approaches have been tested. For example, the overexpression of a gene for vacuolar iron sequestration, *AtV1T1*, resulted in altered partitioning of iron, with an iron content that was three to seven times higher in storage roots in transgenic plants compared to the wild type in field trials. The coexpression of a mutant *Arabidopsis thaliana* iron transporter *IRT1* and *A. thaliana* ferritin (*FER1*) produced transgenic cassava plants that accumulated iron levels that were 7–18 times higher and zinc levels that were 3–10 times higher, providing 40–50% of estimated average requirements (EAR) of iron and 60–70% of EAR of zinc for 1–6-year-old children and nonlactating, nonpregnant West African women [50]. In recent developments in the genomics of cassava, a haplotype-resolved diploid genome of an African landrace cassava (‘TMEB 117’) has been sequenced to a high level of accuracy providing valuable insights into the heterozygous genome of cassava and its resistance to African cassava mosaic virus [51].

Cassava mosaic disease (CMD), caused by a group of at least eight geminiviruses transmitted by white fly *Bemisia tabaci* and through infected planting material, is the most devastating disease of cassava in Africa and the Indian subcontinent. With an annual estimated economic loss of USD1.9–2.7 billion, it is considered the most damaging plant virus disease in the world [52]. The newly emerged cassava brown streak disease (CBSD) caused by two species of ipomoviruses, Cassava brown streak virus (CBSV) and Ugandan cassava brown streak virus (UCBSV), also transmitted by white fly, has become a serious threat to millions of subsistence farmers in Eastern and Central Africa. RNAi-based technology can be deployed for the simultaneous management of multiple viruses using hairpin probes with sequences from several viruses. This approach was used by Beyene et al. [53] to develop transgenic plants of the popular African cultivar ‘TME204’, expressing an inverted repeat construct derived from coat protein sequences from CBSV and UCBSV fused in tandem. The resulting transgenic plants showed robust resistance to both viruses while retaining the desirable agronomic characteristics of the cultivar preferred by Ugandan farmers, ‘TME204’ [53]. CBSD-tolerant GM cassava was approved for cultivation in Kenya in 2020 [54]. Although the disease resistance and safety of the cultivar has been tested, the release is still surrounded by skepticism and criticism [55]. A non-GM approach to mutation induction has also been attempted to develop mutants with a tolerance for CMD and CBSD. Field trials conducted in different agro-ecological regions in Kenya have revealed that the three mutants have better tolerance to these diseases than their respective parents [56]. On the other hand, an attempt to increase β carotene content by co-expression of transgenes for deoxy-d-xylulose-5-phosphate synthase and bacterial phytoene synthase in cassava resulted in reduced dry matter and starch content, despite a 15–20-fold increase in carotenoids [57]. 

Sweet potato (*Ipomoea batatas* (L.) Lam—Convolvulaceae) is the other most important root and tuber crop cultivated worldwide, ranking seventh overall in terms of production [57] and having considerable potential to reduce the Global Hunger Index, particularly in sub-Saharan Africa, the Pacific Islands, and parts of Asia [58]. Hexaploidy and self- and cross-incompatibility in sweet potato introduce difficulties in using both traditional breeding and genomic approaches for their improvement. Nevertheless, in recent times, next-generation sequencing, high-throughput genotyping, and phenotyping technologies have been applied to this crop, providing genomic tools and resources for its genetic improvement. The available genomic resources, databases, bioinformatic tools, and the current reference genome of sweet potato were recently reviewed by Yan et al. [46]. The improvement of sweet potato can now be fast-tracked thanks to the availability of efficient Agrobacterium transformation systems based on embryogenic suspension cultures [59] and via direct organogenesis using petiole explants [60], enabling, for example, the development of transgenic sweet potato with herbicide tolerance [61]. Biolistic transformation has also been successfully developed for this species [62]. Sweet potato feathery mottle virus (SPFMV), a *Potyvirus* in the family *Potyviridae*, is a devastating virus for sweet potato growers worldwide. Using the electroporation method of transformation, Okada et al. [63] introduced an expression vector harboring the coat protein of SPFMV and hygromycin phosphotransferase genes driven by cauliflower mosaic virus 35 S promoter into a popular sweet potato variety, ‘Chikei 682-11’. Greenhouse testing of three independent transformants showed resistance to both primary and secondary infection by the virus, confirming the possibility of using coat-protein-mediated resistance to SPFMV [63]. 

Potato, cassava, and sweet potato, the three most important root and tuber crops worldwide, are featured in this Special Issue. Traditional hybridization-based potato breeding is cumbersome due to the tetraploid nature of cultivated potato (*Solanum tuberosum*) and its narrow genetic base. Starch content on a fresh and dry weight basis is an important breeding objective in potato breeding. Despite the common use of in vitro-produced microtubers in commercial production and germplasm conservation of potato, in vitro techniques remain in limited use as research tools for understanding the biochemical and molecular bases of the physiology of tubers or in breeding. Traits such as dormancy [64,65], cold-induced sweetening [66], and salinity tolerance [67] have been shown to be amenable to examination when using this system. In their paper published in this Special Issue, Adley et al. (contribution 10) used callus cultures to induce somaclonal variation in the variety ‘Lady Rosetta’ and screened 105 regenerants for starch content. They isolated a somaclonal variant with 42% and 61% higher fresh and dry weights, respectively. This somaclone had a 10% and 75% higher starch content based on the dry weight and average content per plant, respectively, compared to ‘Lady Rosetta’. Molecular analysis using real-time PCR of the new variant named ‘Ros 119’ demonstrated upregulation of six starch-synthesis genes (contribution 10).

Cassava (*Manihot esculenta* Crantz) is the third largest source of food carbohydrates in the tropics after rice and maize. It is one of the most drought-tolerant food crops; hence, it is the staple crop in the poorest and most remote areas in Africa. With great variation in climatic conditions in the tropics, particularly with regard to rainfall and temperature, cultivars with stable high yields across environments are required, especially in large countries with varying climates. In this Special Issue, Amelework et al. (contribution 11) report the results of testing 11 advanced selections of cassava in six sites across South Africa. They analyzed of genotype and environment, and the effects of their interaction on fresh root yield (FRY) and dry matter content (DMC). The results revealed that the variation in percentage due to genotype x environment interaction was highest for FRY, whereas genotypic variation was the main contributor to the total variation in DMC. The authors identified two genotypes providing high DMC and FRY across all environments, and three sites that are representative of the variation in climatic conditions, suitable for variety evaluation and breeding.

Sweet potato (*Ipomoea batatas* (L.) Lam.) is a valuable source of carbohydrates, vitamins, fibers, and minerals, and is considered one of the most important crops in both tropical and subtropical climates. *Cylas formicarius* (Fabricius) and West Indian sweet potato weevil (*Euscepes postfasciatus* (Fairmaire)) are the most damaging pests of sweet potato in many continents including Central and South America, the South Pacific, and Japan. In the first comprehensive gene expression analysis during weevil infection in the resistant ‘Kyushu No 166’ cultivar published in this Special Issue, Nokihara et al. (contribution 12) show that genes related to phosphorylation, metabolic, and cellular processes, as well as terpenoid-related genes responsible for producing plant-derived juvenile hormones, are upregulated.

## 5. Industrial Crops

The only plantation crop that sustainably supplies natural rubber for aviation and other industries, as well as domestic uses, is the Pará rubber tree (*Hevea brasiliensis*); this tree originated in the Amazon, but was domesticated in Asia. As a result of domestication in a distant continent, Pará rubber tree populations have a very narrow genetic base in cultivation and are prone to many diseases. With 3–4 years from seed planting to flowering, 6–7 years to start tapping for rubber, and another 5–10 years required to assess yield, traditional breeding is a difficult and prolonged process. It takes, on average, three decades to complete the entire cycle of selection and release of new clones for planting. Therefore, marker-assisted selection and genetic transformation can accelerate breeding. Somatic embryo-based transformation has been developed for *Hevea*, and the advances made in this area have been discussed in detail in a recent review by Wang et al. [68]. The first draft of the *H. brasiliensis* genome was reported by Rahman et al. [69] in 2013. Their results indicated that 78% of the genome comprised repetitive DNA and 12.7% of the gene models unique to *Hevea*. Key genes associated with rubber biosynthesis, disease resistance, and allergenicity were identified [69]. Genome assembly of the popular rubber clone ‘RRIM 600’ revealed an expansion in the number of rubber-biosynthesis-related genes and their high expression in latex, explaining its high rubber yield [70]. This was further confirmed in the report by Tang et al. [71], who demonstrated the expansion of the *REF/SRPP* (rubber elongation factor/small rubber particle protein) gene family and its divergence. Using a high-density single-nucleotide polymorphism (SNP)-based map, Pootakham et al. [72] were able to anchor about two thirds of protein-coding genes into 18 linkage groups of the *H. brasiliensis* ‘BPM 24’ clone. Comparative analysis of the intragenomic homeologous synteny provided evidence for the presence of paleotetraploidy in the species. Chao et al. [73] demonstrated the relationship of increase in rubber yields during the domestication process with the increase in the number of laticifer rings and its high correlation with *HbPSK5* encoding the small-peptide hormone phytosulfokine—a key domestication gene of rubber. Thus, through genomic studies, our understanding of the expression of different traits of agronomic interest in rubber trees has increased. In a recent review, Priyadarshan [74] discussed the possible application of molecular markers to rubber plants in their juvenile phase to select for traits expressed after maturity using genomic selection. These studies will no doubt accelerate the breeding of new rubber clones with desired traits and improve the efficiency of breeding as well.

The three main diseases affecting rubber plantations worldwide are caused by *Phytophthora* spp. (causing shoot rot, abnormal leaf fall, patch canker, and black stripe diseases), *Corynespora cassiicola* (causing *Corynospora* leaf fall disease), and *Colletotrichum* spp. (causing *Colletotrichum* leaf fall disease). All these diseases reduce plant growth and latex yield, and are controlled using fungicides. Breeding for resistance using traditional hybridization and selection is practically impossible because of the high degree of heterozygosity in Pará rubber clones, thus requiring several backcrosses to introgress genes controlling disease resistance in this species with a long breeding cycle and the large land area required for screening such populations. Thus, early screening of breeding populations at the seedling stage can revolutionize breeding of this valuable species. Polymerase chain reaction (PCR) is a simple and rapid method that can detect nucleotide polymorphisms and sequence variation. When PCR reactions are conducted competitively in the presence of allele-specific primers to preferentially amplify only certain alleles, the variant is called allele-specific PCR (AS-PCR). Kompetitive Allele-Specific PCR (KASP) is a variant of AS-PCR modified for fluorescence-based detection of amplification results. In this Special Issue, Roy et al. (contribution 13) report the identification of 12 single nucleotide polymorphisms (SNPs) significantly associated with resistance against *Phytophthora*, *Corynespora,* and *Colletotrichum* in six linkage groups using an integrated linkage map of a F_1_ progeny in an interspecific cross between *H. brasiliensis* (‘RRII 105’—susceptible parent) and *H. benthamiana* (‘F4542’—resistant parent) using 23,978 markers. To demonstrate the possible application of these findings in marker-assisted breeding of rubber for resistance to these diseases, the authors used KASP assays for all 12 SNPs that showed significant associations with the disease traits. When the KASP assays were applied to 178 ‘RRII’ 105 × ‘F4542’ F_1_ progeny, the genotypes could be clearly separated on the basis of resistance. Four F_1_ plants were found to carry favorable alleles from *H. benthamiana* for all the three disease traits. They also predicted 41 key genes within proximity to those SNPs that were previously reported to be associated with disease resistance. This is the first report of the development of molecular markers for the three diseases, and this work has the potential to fast-track the breeding of disease-resistant Pará rubber.

## 6. New Crops for Arid Regions

The impact of climate change on the agro-forestry systems and the adaptive capacity of plants and animals will be of strategic importance in the immediate future to ensure food security. Numerous evidence suggests that reduced water availability and rising temperatures associated with global warming will have a significant impact on agriculture in the future [75]. Water is an essential component of agricultural production. According to UN and FAO data, approximately 3000–5000 L of water are needed to meet the daily food requirement of a person [76]. Furthermore, in the Global Risks Report of the World Economic Forum, water crises are stated as the third most important global risk in terms of impact on humanity [77].

Climate change has caused an increase in average temperatures and an ever-increasing demand for water. Furthermore, given that the demand for food production is likely to increase in the future [78], the challenge of sustainably producing food and non-food resources with organisms adapted to new environmental conditions will become of strategic interest. The application of biotechnology to drought-resistant crops would be a long-term solution for the production of more food with less water in increasingly warmer environments. An important contribution to achieving this objective comes from the use of cacti, known for their minimum water requirement; they have been grown extensively in arid lands, for food, feeds, and medicinal and therapeutic uses [79]. Cacti utilize Crassulacean Acid Metabolism (CAM) for photosynthesis, a unique process in desert plants that opens their stomata only at night when the plant is relatively cool, so that less moisture is lost through transpiration. Among the most interesting species, *Opuntia ficus-indica* (commonly known as prickly pear) represents an archetypal constitutive CAM species. In this Special Issue, Carra et al. (contribution 14) describe the use of the in vitro rescue of zygotic embryos for the genetic improvement of *O. ficus-indica*. Prickly pear cactus is an important forage and food source in arid and semiarid ecosystems, and is the most important cactus species cultivated globally. Both fruits and seeds have shown important antioxidant and nutritional properties, and can be a potential source of functional and nutraceutical ingredients. This crop is one of the most promising in the face of new environmental conditions due to climate change which will increasingly reduce the availability of water. In fact, it is able to produce fruits even in conditions where other crops cannot survive. The high degree of apomixis in the species is a hindrance in plant breeding programs where genetic segregation is sought for the selection of superior genotypes. Therefore, the protocols described for in vitro embryo rescue open a pathway to increase the availability of zygotic seedlings in *O. ficus-indica* breeding programs through in ovule embryo culture.

## 7. Ornamental Crops

**Figure 3:**
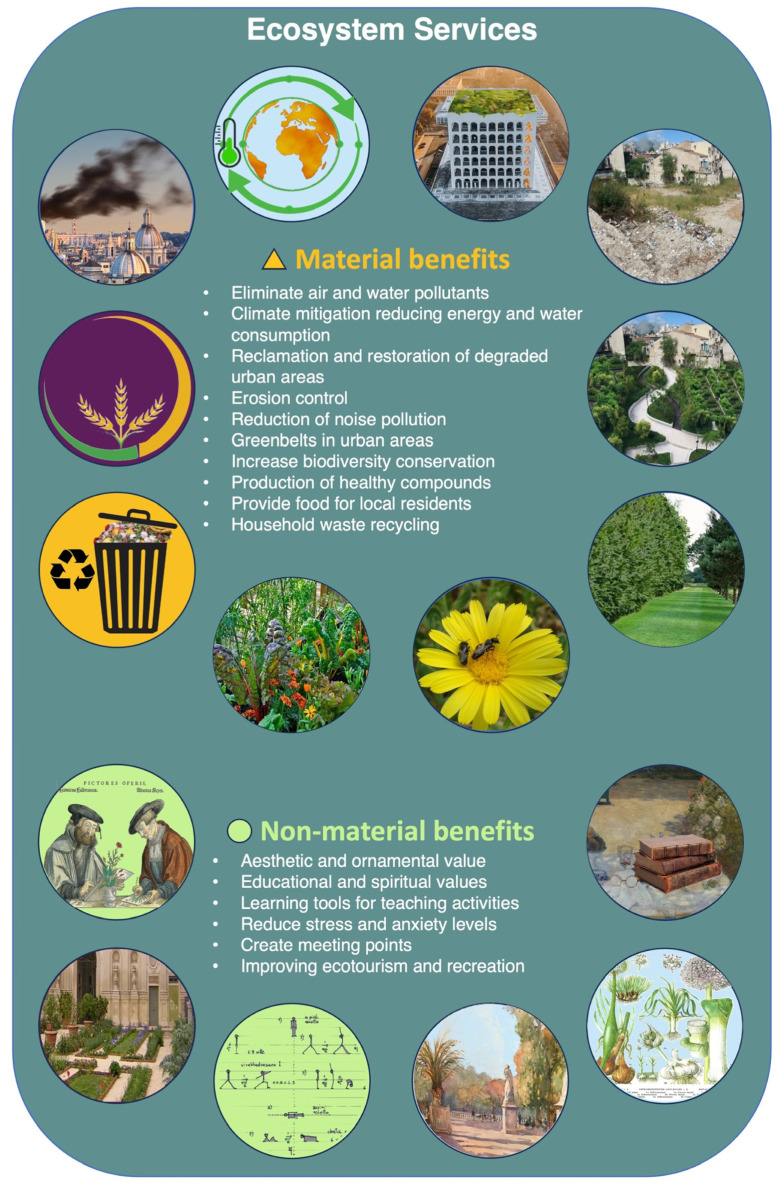
Ecosystem services and benefits obtained from ornamental plants in an urban and peri-urban area.

The economic importance of ornamental plants has been increasing significantly in many countries with international demand expanding rapidly, providing many benefits to nature and humans both in urban and peri-urban areas. Ornamental plants, including cut flowers, foliage, and live plants, showed a positive trend in export growth, which led to an aggregate world value of around EUR 18 billion in 2020 [80]. Ornamental plants, cultivated both in indoor and outdoor environments, can contribute to human health and wellbeing, and can ensure essential environmental services (Figure 3), including the mitigation of the climate change, reduction in air and soil pollution, and providing food for habitants [81,82]. 

Ornamentals are a hugely diverse group of commercially significant plants that are grown and traded usually for decorative purposes, either as whole plants or for their parts. Among the ornamental plants, orchids have a special place due to their stunning displays of color and the shape of the flowers. Apart from their scientific fascination due to many unusual biological features, orchids account for a great part of the global floriculture trade. These are either traded as whole plants or cut flowers. Novelty is of great importance in the ornamental plant industry and many biotechnological approaches have applications in developing such novelties. In this Special Issue, such applications in two valuable orchid species of commercial importance have been described.

Polyploidy is much more pronounced in the plant kingdom than in the animal kingdom and has played a key role in plant evolution, species adaptation, and spread. Polyploids are more frequent among agricultural crops than in nature as they have many agronomic benefits such as larger size of organs, higher concentration of secondary metabolites, and better adaptation. Although polyploidization occurs in nature sporadically, in plant breeding it is artificially induced at higher frequencies. The development of new polyploid orchids often results in superior ornamental characteristics compared to their diploid counterparts. Orchids of the genus *Cattleya* are commercialized as hybrids. Although there are protocols for the polyploidization of *Cattelya* spp., there are no protocols for their interspecific or intergeneric hybrids, the widely commercialized types. In the current Special Issue, Vilcherrez-Atoche et al. (contribution 15) describe the use of in vitro cultured protocorms and seeds to induce polyploidy in *Brassolaeliocattleya,* a cross between three genera: *Brassavola*, *Laelia*, and *Cattleya* [83], using colchicine—an inhibitor of microtubule formation in the chromatic spindle resulting in the nondisjunction of chromosomes; this, in turn, results in the duplication of chromosomes within the nucleus. They report higher rates of polyploidization in protocorms and use flowcytometry to confirm the ploidy level in regenerants.

Dendrobium orchids are traded both as cut flowers and potted plants. They are among the top ten orchid taxa of commercial importance, with a wide variety of choices in flower color, texture, and shape, and a good vase life. However, two varieties account for 70% of the world trade, indicating a limited choice of varieties suited for the export market. Hence, there is an enormous potential for the right cultivars to break into the export market. However, compatibility barriers in intersectional crossing, negative genetic linkages in promising traits and prolonged juvenile phase and high mortality at hardening stages of in vitro cultures are barriers to improvement and commercialization of novel Dendrobiums. Induced mutations offer unique opportunities to improve elite cultivars by rectifying a defect such as late flowering, disease susceptibility or flower size [23]. The Dendrobium hybrid ‘Emma White’ is popular, but has a long juvenility after micropropagation. With the objective of developing an early flowering mutant of ‘Emma White’, Sherpa et al. (contribution 16) used gamma irradiation on protocorm-like bodies of this mutant and studied growth responses at different dosages and found optimal dose levels for producing high mutation rates with low mortality. By screening the mutant population, they were able to isolate a mutant with early flowering (294 days vs. 959 days in “Emma White’), demonstrating the value of in vitro mutagenesis in improving orchids.

## 8. Development of New Methodologies in Plant Biotechnology

Genetic transformation is a breakthrough biotechnology that has transformed agriculture in recent times, with 29 countries growing over 190.5 million ha of biotech crops. Although the number of countries is small, the impact of biotech crops is high, with most of North and South America, China, India, Australia, Indonesia, Vietnam, Myanmar, Pakistan, Bangladesh, the Philippines, and large countries in Africa (South Africa, Nigeria, Ethiopia, Kenya, Sudan, etc.) growing these crops. Thanks to improved traits such as insect resistance, growing these crops is more environmentally friendly, and the additional income from these crops is estimated at USD 225 billion for the 20-year period since 1996 thanks to the production of an additional 824 Mt of food, feed, and fiber [6]. Stable gene transformation systems and strong positive selection markers are imperative for developing transgenic plants. Co-cultivation of the host plant tissue in vitro along with Agrobacterium carrying the desired gene construct is the traditional method for transformation, and antibiotic- or herbicide-resistant genes inserted along with the desired gene/s are used as positive selection markers. Due to environmental and health concerns with such genes in plants, other markers such as β-glucuronidase or fluorescent protein markers are used, but they require destructive staining for former or expensive equipment to detect fluorescent cells for the latter option. Therefore, more robust and simple selection marker development for crop transformation is important. In this Special Issue, Lim et al. (contribution 17) report the development of a simple system for the selection of transgenic plants. They report the use of the R2R3 MYB transcription factor gene *CaAN2* from chili pepper (*Capsicum annuum*) for use as a visible selection marker with successful selection in both transient assays and in stable transformation, using tobacco as the model system. Transgenic tobacco plants harboring the chili pepper *CaAN2* readily promoted the accumulation of anthocyanin throughout the plant, allowing easy selection at the plant regeneration stage of the transformation experiment without the involvement of additional steps to identify the transgenic plants. The method has the potential to dramatically improve the efficiency of selection in plant genetic transformation, a key biotechnological approach for crop improvement.

## Abbreviations

**Abbreviations**	**Full Name**
AS-PCR	allele-specific PCR
Cas	Crispr associated protein
CAM	Crassulacean Acid Metabolism
CBSV	Cassava Brown Streak Virus; CMD—Cassava Mosaic Disease
CRISPR	Clustered Regularly Interspaced Short Palindromic Repeats
DMC	Dry Matter Content
DXS	Deoxy-d-xylulose-5-phosphate synthase
FAO	Food and Agriculture Organization of the United Nations
FRY	Fresh Root Yield
KASP	Kompetitive Allele-Specific PCR
PCR	Polymerase Chain Reaction
PR	Perennial Rice
QTL	Quantitative Trait Loci
SNP	Single-nucleotide Polymorphism
SPP	Single Plant Progeny
TALENs	Transcription Activator-Like Effector Nucleases TILLING—Targeting Induced Local Lesions in Genomes
UNGA	United Nations General Assembly
ZFN	Zinc Finger Nucleases
